# Improvement effects of transplanting pancreatic islet that previously incubated with biomaterials on the diabetic nephropathy in STZ- diabetic rats

**DOI:** 10.1186/s12882-024-03572-4

**Published:** 2024-05-09

**Authors:** Marzieh Nemati, Zahra Hosseinzadeh, Fatemeh Nemati, Farhad Koohpeyma

**Affiliations:** 1 Endocrinology and Metabolism Research Center, Shiraz University of Medical Sciences, Shiraz, Iran; 2 School of Dentistry, Shiraz University of Medical Science, Shiraz, Iran

**Keywords:** Diabetic nephropathy, Platelet rich plasma, Pancreatic islet homogenate, Biomaterials, Islet transplantation, Stereological changes, Oxidative stress

## Abstract

**Background:**

Islet transplantation is an effective treatment for diabetes or even its complications. Aim of this study is to investigate efficacy of biomaterial treated islet transplantation on treating diabetic nephropathy.

**Methods:**

Male rats were randomly divided into 6 groups; Control, diabetic control, diabetic transplanted with untreated islets, with platelet rich plasma treated islets, with pancreatic islets homogenate treated islets, or with these biomaterials combination treated islets. Islets cultured with biomaterials and transplanted to diabetic rats. After 60 days, biochemical, oxidative stress, and stereological parameters were assessed.

**Results:**

Serum albumin and BUN concentration, decreased and increased respectively, Oxidative stress of kidney impaired, kidney weight, volume of kidney, cortex, medulla, glomerulus, proximal and distal tubules, collecting ducts, vessels, inflammatory, necrotic and fibrotic tissue in diabetic group increased compared to control group (*p* < 0.001). In treated groups, especially pancreatic islets homogenate treated islets transplanting animals, there was significant changes in kidney weight, and volume of kidney, proximal and distal tubules, Henle’s loop and collecting ducts compared with diabetic group (*p* = 0.013 to *p* < 0.001). Combination treated islets animals showed significant increase in vessel volume compared to diabetic group (*p* < 0.001). Necrotic and fibrotic tissue significantly decreased in islets treated than untreated islet animals, it was higher in pancreatic islets homogenate, and combination treated islets groups (*p* = 0.001).

**Conclusions:**

Biomaterials treated islets transplanting could improve diabetic nephropathy. Improvement of oxidative stress followed by controlling glucose level, and effects of growth factors presenting in biomaterials can be considered as capable underlying mechanism of ameliorating inflammatory, necrotic and fibrotic tissue volume.

## Introduction

Diabetic nephropathy (DN) is very common in both type 1 and type 2 diabetic patients [1, 2] and can be noted from onset to the last stage of diabetes [3]. Pathophysiological changes in DN hallmarks include glomerular basement membrane GBM thickening, glomerular hypertrophy, mesangial expansion, and extracellular matrix deposition in the renal mesangium and tubulointerstitial regions [4, 5]. That decrease renal function and eventually lead to end-stage renal kidney disease (ESRD) [6, 7]. ESRD reduces the quality of life and survival of diabetic patients [8]. Chronic hyperglycemia is not only the main cause of renal injury but is also involved in the development of diabetic nephropathy [9]. Accordingly, controlling and reducing plasma glucose levels is the main goal of diabetic therapeutic strategies.

Various therapeutic options for diabetes and its related complications have been used, such as insulin therapy, pancreatic transplantation, and pancreatic islet transplantation [10], but increasing the number of progressive DNs demonstrated that none of these therapeutic methods were sufficiently effective in eliminating diabetes and its complications. Therefore, finding a new strategy or improving the existing method for controlling or reversing DN is essential.

Islet transplantation has been proven to be very effective in lowering hyperglycemia, managing diabetes, or reversing it. Despite this, there are only a few studies about the beneficial effects of islet transplantation regarding controlling and even avoiding diabetic complications. These complications include waning clinical features of diabetes, averting the deterioration of renal insults from diabetic nephropathy, and sometimes reversing them [11–13]. The results of the other study demonstrated that islet transplantation may prevent the progression of testicular damage by downregulating oxidative stress and inhibiting inflammation pathways [14]. As mentioned before, effectively managing and reducing blood sugar levels plays a role in mitigating the kidney problems linked to diabetic nephropathy. Therefore, any treatment method that leads to a decrease in blood sugar has shown results in treating nephropathy.

Previous studies demonstrated that transplantation of islets previously treated with biomaterials (Platelet-rich plasma (PRP) or pancreatic islet homogenate (PIH)) could significantly decrease blood glucose [15–17]. PRP, a biomaterial, contains varied growth factors that stimulate different signaling pathways and are essential for processes such as proliferation, differentiation, collagen, and matrix production [18]. PRP is safe and used effectively in different fields [19] due to its easy handling and lack of immune response in the recipient [15, 16]..

PIH, another used biomaterial, includes different proteins and growth factors such as collagen, HGF, and VEGF [17]. PIH is considerably safer in islet transplantation because it is more adaptable in texture, immunity, and cellular mechanisms in comparison with other synthetic materials and also more reasonable due to the fact that unsuitable isolated islets for transplantation were homogenated and used as a supplementary biomaterial [18].

Based on past studies, the transplantation of biomaterial-treated islets might improve the success of transplant outcomes and diminish blood glucose [18–20]. Therefore, this study focuses on investigating whether biomaterial-treated islet transplantation can prevent kidney changes in diabetic nephropathy.

## Materials and methods

### Study design and animal

42 male Sprague-Dawley rats (250–280 g) were obtained from the stock of rats bred in the animal house of the Research Institute of Shiraz University of Medical Sciences (Shiraz, Iran). Animals were housed in 2/cages under standard conditions (temperature 24 ± 2 °C and relative humidity 45%, 12-h light cycle) with free access to food and water. The rats were randomized into six groups (7 in each group): Control (healthy control rats), Diabetic control (untreated diabetic rats), IT (Diabetic rats were transplanted with 400 untreated islets), IT-PRP (Diabetic rats were transplanted with PRP-treated islets: 400 islets that previously incubated with 1 ml RPMI containing 10% PRP, platelet; 1500 $\times$10^3^/µl for 24 h), IT-PIH (Diabetic rats were transplanted with PIH-treated islets: 400 islets that previously incubated with 1 ml RPMI containing 10% PIH, protein; 100 µg for 24 h) and IT-PRP&PIH (Diabetic rats were transplanted with a PRP& PIH-treated islets: 400 islets that previously incubated with 1 ml RPMI containing 10% PRP, platelet; 1500 $\times$10^3^/µl + 10% PIH, protein; 100 µg for 24 h). To avoid wound infection after transplantation surgery, daily cage cleaning was performed rigorously.

### Diabetes induction

Diabetes mellitus was induced by a single intra-peritoneal (i.p.) injection of STZ (65 mg/kg, i.p.; Sigma, USA), which was dissolved in citrate buffer (pH 4.5) immediately before use. Blood glucose was measured by using a glucometer (Accutrend Plus; Roche, Mannheim, Germany) from the tail vein sampling. The rats were accepted as diabetic when they exhibited hyperglycemia (blood glucose levels > 350 mg/dl) and were used for transplantation studies.

### Platelet-Rich plasma preparation

For preparing platelet-rich plasma, rats were anesthetized with ketamine and xylene (50/10 mg/kg), whole blood was collected through cardiac puncture and drained into a 15 ml tube containing anticoagulant (3.2% sodium citrate with 9/1 ratio blood to sodium citrate), centrifuged 2 times, first at a speed of 1000 rpm for 15 min, followed by a second step at a speed of 3000 rpm for 5 min. The lower 1/3 layer of remaining liquid is PRP [19].

On average, each rat yielded about 6 ml of blood specimens. Approximately 55% of this blood was plasma (3300 µl), the lower third of the remaining liquids after the second centrifugation (1100 µl), was PRP.

In both in vitro and in vivo, experimentation involved two distinct groups incorporating PRP: Islet-PRP, Islet-PRP&PIH, and IT-PRP, IT-PRP&PIH: 7 rats/ group. Considering that 100 µl PRP is required for assessing in vitro parameters before transplantation (islet function, viability) and for in vivo islet transplantation of each rat, a total of 5000 µl PRP (in vivo: 1400 and in vitro: 3600 µl) were used, all together 5 rats were used for preparing PRP.

### Pancreatic islet homogenate preparation

For preparing pancreatic islet homogenate, after anesthesia with Ketamine/ Xylazine (50/10 mg/kg), laparotomy was performed. The end of the common bile duct that enters the duodenum was clamped, then polyethylene cannula containing 10 ml of cold Hanks buffer and collagenase P (Collagenase P Roche, Cat. # 11 213 865 001, Mannheim, Germany, 0.5 mg/ml) was inserted to duct. Then the inflated pancreas was removed and after cleaning from non-pancreatic tissue, incubating in the water bath (17 min, 37˚ c), shaking the digested pancreas, and purifying islet from non-islet tissue, the islets were isolated by handpicking under stereomicroscope. After adding lysis buffer to an unsuitable pancreatic isolated islet for transplantation (including breakage of the islet membrane, islet with rough edge, very small or large islet), they were homogenated with an ultrasonic homogenizer (Bendelin prob MS72 HD-3100), and then centrifuged. The prepared supernatant was PIH [18].

As described for PRP, there are two groups containing PIH in both in vitro and in vivo experiments: Islet-PIH, Islet-PRP&PIH, IT-PIH, and IT-PRP&PIH. For each of them 100 µl of PIH containing 100 µg of protein (a total of 5000 µg protein) was needed and used. By islet isolation of each donor rat provided an average of 400 isolated islets, out of which nearly 50 islets were unsuitable for the transplant (such as torn or have not smooth edge) and used to prepare PIH. The amount of protein extracted from 50 unsuitable islets was approximately 984 µg/ml, and 300 islets was used to prepare the required amount of protein. In other words, 6 rats were used to prepare PIH.

### Culture of isolated islets

Isolated islets from Sprague–Dawley rats before transplantation were cultured in 1 ml RPMI- 1640 media, or RPMI-1640 media supplemented with 10% PRP (900 ml RPMI+100 ml PRP, plt: 1500$\times$10^3^/ml), or RPMI-1640 media supplemented with 10% PIH (900 ml RPMI+100 ml PIH, pro: 100 *µ*g), or RPMI-1640 media supplemented with 10% PRP &PIH, (800 ml RPMI+100 ml PRP, plt: 1500$\times$10^3^/ml + 100 ml PIH, pro: 100 *µ*g) and incubated at 37 _C in 5% CO2 and 95% air for 24 h [20].

### Assessment function and viability of biomaterials–treated islet

Because the viability and function of islets are damaged in both pre-transplantation (digestion and culture) and post transplantation due to multiple factors, including loss of vasculature, environmental disruption, and oxidative stress. Viability, function and oxidative stress markers of isolated islets were evaluated before transplantation to ensure that the isolated islets are functional and viable. At the end of this 24-hour incubation of the islets with or without biomaterials, the quantification of secreted and content of insulin was measured using an enzyme-linked immunosorbent assay method (ELISA and Mercodia in Upsala, Sweden).

Viability of the isolated islets was assessed using Annexin V (Life Technologies Japan– Tokyo) and Propidium Iodide fluorescent dyes (Sigma-Aldrich, St. Louis, MO) [19, 20].

Also, post transplantation islet functionality and quality was assessed by reversing diabetes and hyperglycemia in islet transplanting rats [20].

### Islet transplantation

Animals were anesthesia with Ketamine/ Xylazine (50/10 mg/kg). Aliquots of 400 islets equivalent (IEQ) that were cultured with or without PRP and PIH for 24 h, were aspirated into a polyethylene tubing P-50 (Harvard Apparatus, Holliston, MA, USA) and placed on ice. The recipient animals were anesthetized with ketamine and xylene (50/10 mg/kg), a small incision was performed on the left flank site, The kidney was exposed, and islets were transplanted under the capsule of the left kidney as already reported [18, 19].

### Blood sample collection

When the trial came to an end, exactly 60 days after the islet transplant, rats were anesthetized with ketamine and xylene (50/10 mg/kg) and blood samples were collected by cardiac puncture for the assessment of serum glucose, BUN, Cr, and Uric acid.

### Kidney tissue collection

Both kidneys were removed at the conclusion of the trial, for the assessment of stereological parameters and the measurement of oxidative stress markers in all experimental animals. It should be noted that the samples were numbered, and the group of animals was not mentioned and was done blindly.

### Measurement of oxidative stress markers

The right kidney was used to evaluate antioxidant enzyme activity. Lipid peroxidation. Malondialdehyde (MDA) levels were measured using the thiobarbituric acid reactive substances (TBARs) method. Superoxide dismutase (SOD), Glutathione Peroxidase (GPx) activity [19] were computed by commercial Assay Kits (ZellBio GmbH, Ulm, Germany) using the colorimetrical method, and NOx (nitrite/ nitrate) by using the modified Griess method [21]. It should be noted that the samples were numbered, and the group of animals was not mentioned and was done blindly.

### Tissue preparing for stereological assessment

**Fig. 1:**
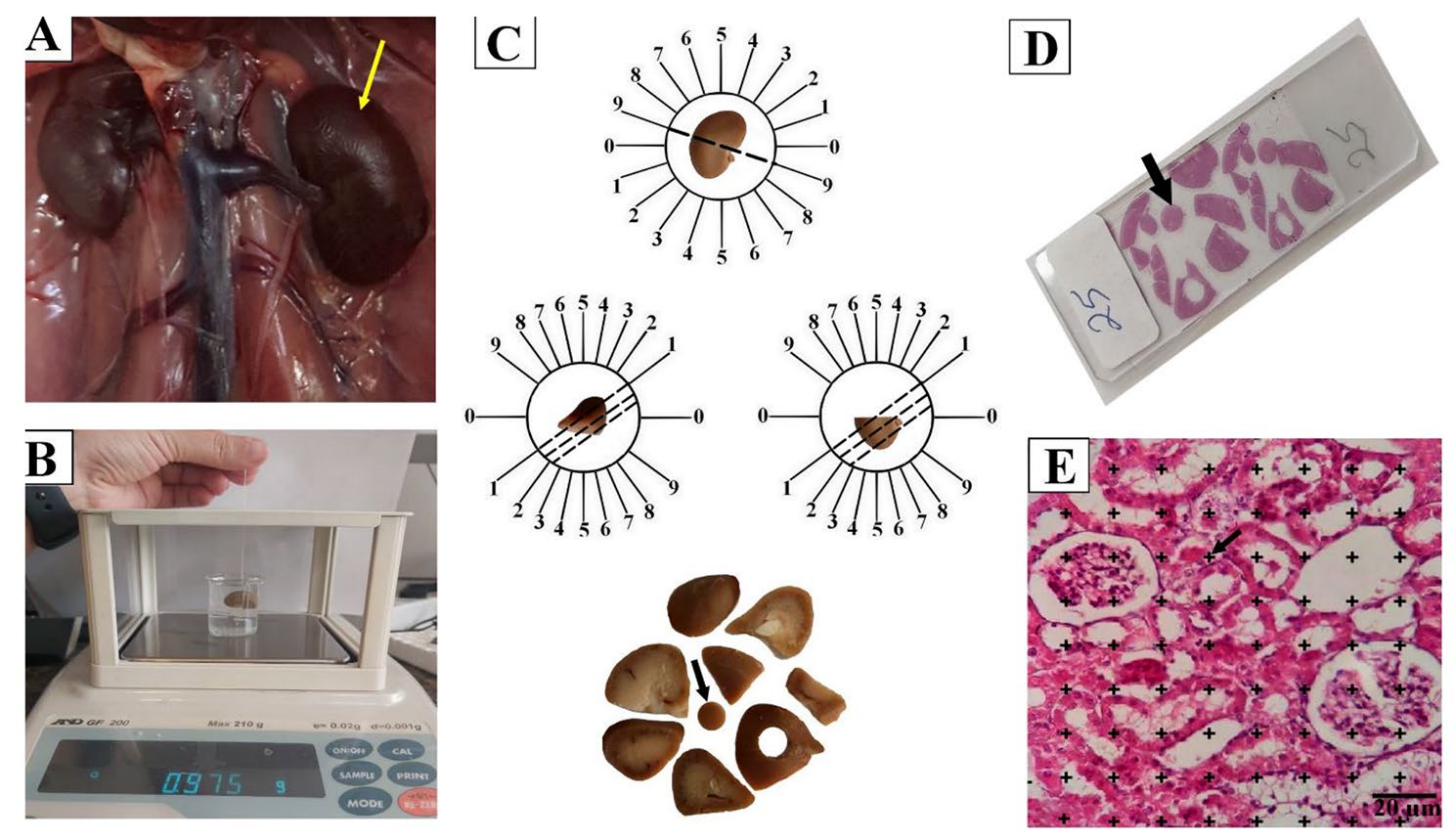
left kidney (yellow arrow) was removed (**A**), total volume was measured by immersion method (**B**), oriented method used to isotropic uniform random (IUR) sectioning, kidney was sectioned into 8–12 parallel slabs, and a circle was take from slabs using a trocar (arrow) for estimating shrinkage (**C**), cut surface of slabs was stained by tow staining method (**D**), by use of point grade method (arrow), stereological parameters was evaluated. Point counting Method (The accepted points that hit the right upper corner of each target’s cross were considered) (E)

Upon the culmination of experiment, that is, Fig. 1, shows the surgical incision that was made after administering anesthesia. In this controlled state, the left kidney was removed, and its weight was recorded (Fig. 1A). Subsequently, the primary volume of the kidney was determined using an immersion method (Fig. 1B). All tissues were fixed in a formalin buffer. Isotropic uniform random (IUR) sections were created using the orientor method with the aim of having the best outcomes for completen preparation. The kidney was placed on a circle-shaped area, and then an arbitrary number between 0 and 9 was picked (here it was 9). In addition, the kidney was extensively cut down into two equal halves through the process of thorough sectioning. Then, the cut surfaces were positioned in a manner compatible with the orientation indicated by the random number (in this case, it was zero). The last random number used was to divide the kidney into two sections for a second time. Samples from these sections were obtained and rolled into a troca sample (Fig. 1C) to find out any possible shrinkage [22]. Thereafter, the tissue sections were placed in paraffin frames and stained again using “Masson’s trichrome” and “Hematoxylin eosin” dyes, which will later be subjected to stereological analysis (Fig. 1D). The preparation of thin Sect. (5 micrometers) and staining processes were involved. Lastly, several stereological parameters were evaluated using the point counting method, in which only the valid points that cut into the top right corner of each target’s cross was counted ( Fig. 1E). To assess the shrinkage amount and the final volume of kidney, the following formula was used:

Degree of shrinkage = 1-$$ ({\frac{Area after}{Area before})}^{\raisebox{1ex}{$3$}\!\left/ \!\raisebox{-1ex}{$2$}\right.}$$

V_Final_= (1

- Degree of shrinkage) × V_primary_

Each section was assessed and analyzed using a video microscopy system that made of a microscope (E-200, Nikon, Tokyo, Japan) connected to a video camera, a computer, and a monitor to evaluate the parameters. Then the volume density of each structure was measured by using the point-counting method. The volume fraction of the cortex and medulla was measured at a final magnification of ×100 and other parameters at ×400”. The kidney, cortex, medulla, necrotic & fibrotic tissue, glomerulus, proximal convoluted tubule (PCT), distal convoluted tubule (DCT), collecting ducts (CD), henle’s loop, and vessel volume were evaluated by using the following formulas: [22]

$$ Vv\left(structure\right)=\sum _{i=1}^{n}p \left(structure\right)/\sum _{i=1}^{n}\left(reference\right)$$

“${\sum }_{i=1}^{n}p(structure$)” was the number of the test points falling on the targeted structure (here cortex, medulla, necrotic & fibrotic tissue, glomerulus, PCT, DCT, CD, henle’s loop, and vessel), and “${\sum }_{i=1}^{n}p$ (reference)” was the total points hitting the kidney tissue sections (Fig. 1). The following formula was used to estimate the absolute targeted structure volume [22]:

$$ V\left(structure\right)=V\left(total\right)\times Vv\left(structure\right)$$

### Statistical analysis

Statistical data analysis was performed using GraphPad Prism software version 6.0 (Graph-.

Pad Software, La Jolla, CA, USA) and presented as means ± SEM. One-way ANOVA (post hoc: Tukey) was used for multiple comparisons. A value of *p* < 0.05 was considered statistically significant.

## Results

### Islet function and viability assessment before transplantation

The percentage of islet viability in all biomaterial-treated islet groups was significantly higher than the control-Islet group. This difference was more significant in the PRP-Islet and the PRP&PIH-Islet groups (*p* < 0.01). Also, this parameter was higher in the PRP&PIH-Islet than in the PIH-islet group (*p* < 0.05).

Insulin secretion was notably increased in all biomaterial-treated islet groups than the Control-Islet group (*p* < 0.05). Furthermore, insulin content was enhanced in all biomaterial-treated islet groups as compared to the Control-Islet, but this enhancement was significant in the PRP-Islet and the PRP&PIH-Islet groups (*p* < 0.05). Comparison between treated islets showed a marked increase in this parameter in the PRP&PIH-Islet than the PRP-Islet and the PIH-Islet groups (*p* < 0.05) (Table 1).

**Table 1:** In vitro islet function and viability assessment before transplantation

Parameters Groups	Insulin release (ng/ml)		Insulin content (ng/mg protein		Viability (%)		
Control-Islet	1.24 ± 0.16		1.52 ± 0.44^a^		95.54 ± 4.81		
PRP-Islet	2.46 ± 0.42^a^		2.23 ± 0.18^b^		97.25 ± 5.84^a^		
PIH-Islet	2.12 ± 0.39^a^		1.82 ± 0.31^ab^		96.7 ± 5.09^a^		
PRP&PIH-Islet	2.71 ± 0.45^a^		3.61 ± 0.52^c^		98.32 ± 5.46^b^		

Data are presented as mean ± SEM (*n* = 7)

a, b, and c: According to post-hoc Tukey test which was used to make intergroup comparisons, groups with same superscripts were not significantly different

BS: Blood Sugar, ALB: Albumin, BUN: Blood urea nitrogen, Cr: Creatinine

Control-Islet: Untreated islet

PRP-Islet: Islet treated with PRP

PIH-Islet: Islet treated with PIH

PRP&PIH-Islet: Islet treated with PRP&PIH

### Post-transplant outcomes

#### Blood glucose

Table 2 shows that the diabetic animals registered significant higher levels of blood glucose, except for the IT-PRP and the IT-PRP&PIH groups. Besides, all treated animals had much lower serum glucose levels than diabetic and untreated islet transplant animals. For the treated islet transplanting groups, this parameter was considerably lower in the IT-PRP & PIH than in the IT-PIH.

**Table 2:** Effect of biomaterial treated islets transplantation on blood glucose, Albumin, BUN, Cr, and Uric acid content

Parameters Groups	BS (mmol/l)	ALB mg/dl	BUN mg/dl	Cr mg/dl	Uric acid mg/dl
CON	5.29 ± 0.26 ^ae^	4.48 ± 0.44 ^a^	20.26 ± 1.31^a^	0.7 ± 0.00	2.5 ± 0.82
D	14.8 ± 1.12 ^b^	2.5 ± 0.08 ^b^	45.07 ± 2.84 ^b^	0.8 ± 0.02	3.21 ± 0.12
IT	13.16 ± 0.82 ^c^	4.09 ± 0.61 ^ac^	24.48 ± 2.09 ^cd^	0.65 ± 0.01	3.03 ± 013
IT- PRP	6.71 ± 0.43 ^ed^	3.81 ± 0.52 ^c^	27 ± 2.26 ^c^	0.833 ± 0.05	3.83 ± 023
IT- PIH	10.94 ± 0.23 ^d^	4.5 ± 0.71 ^ac^	24.41 ± 2.52 ^d^	0.733 ± 0.03	3.31 ± 0.08
IT- PRP & PIH	5.95 ± 0.29 ^e^	4.35 ± 0.23 ^ac^	26.29 ± 2.26 ^cd^	0.801 ± 0.00	3.72 ± 0.24

Data are presented as mean ± SEM (*n* = 7)

a, b, c, d, and e: According to post-hoc Tukey test which was used to make intergroup comparisons, groups with same superscripts were not significantly different

BS: Blood Sugar, ALB: Albumin, BUN: Blood urea nitrogen, Cr: Creatinine

CON: Healthy control rats

D: Control diabetic rats

IT: Islet transplanted diabetic rats

IT-PRP: Islet transplanted diabetic rats with PRP

IT-PIH: Islet transplanted diabetic rats with PIH

IT-PRP&PIH: Islet transplanted diabetic rats with PRP&PIH

### Biochemical parameters

Serum albumin concentrations, except diabetic control animals, were significantly increased in all islets transplanting diabetic rats and approximately reached the normal level. This change was more marked in IT-PRP&PIH (*p* < 0.0001) and IT-PIH (*p* < 0.0001) than in IT-PRP (*p* < 0.05) and IT (*p* < 0.001) groups. All treated diabetic animals showed a significant increase in serum albumin concentration compare to the diabetic control group.

Following transplantation, serum BUN levels showed a remarkable decrease in all transplanted animals, but still, the concentration of this parameter showed a significant difference compared to the control group. Serum BUN levels were notably lower in all islet transplanting groups than the D group (*p* < 0.001), and among transplanting animals, they were significantly lower in IT-PIH than IT-PRP animals (*p* < 0.05).

Creatinine and uric acid serum concentrations in all groups were in the normal range, and there was no significant difference in the control group (Table 2).

### Oxidative stress markers

Table 3 shows that the MDA concentration of kidney tissues in each group (D (*p* < 0.0001), IT (*p* < 0.05), and IT-PRP (*p* < 0.001)) was significantly higher compared to the control group. On the other hand, SOD activity in the kidney tissues in D (*p* < 0.01) and IT-PRP (*p* < 0.01) groups was lower than in the control group. Compared to the D group, islet transplantation led to a considerable decrease in MDA level (*p* < 0.0001) and elevated (excluding IT-PRP) SOD antioxidant activity (*p* < 0.0.01 to *p* < 0.001). In transplanted animals, treated islets may significantly reduce levels of MDA (*p* < 0.05) and increase the activity of SOD (*p* < 0.05) in comparison to untreated islets transplanted animals. The MDA level was lower (*p* < 0.05) while the SOD activity was higher (*p* < 0.01) in the IT-PIH and IT-PRP&PIH groups compared to the IT-PRP group after comparing treated islet transplanting animals. Diabetic groups, except for IT-PRP&PIH, indicated a pronounced decrease in the level of NOx compared to the control group (*p* < 0.05 to *p* < 0.01) and among islets’ transplanted animals in IT-PIH and IT-PRP&PIH (*p* < 0.05) was significantly higher compared with the IT, as well as in IT-PRP&PIH was remarkably higher than IT-PRP group (*p* < 0.01).

**Table 3:** Effect of biomaterial treated islets transplantation on oxidative stress markers

Parameters Groups	MDA (nmol/mg protein)	SOD (U/ mg protein)	NOx (nmol/ mg protein)
CON	20.95 ± 3.36 ^a^	2.67 ± 0.32 ^ac^	2.3 ± 0.8 ^a^
D	40.46 ± 6.73 ^b^	1.2 ± 0.4 ^b^	1.6 ± 1.2 ^be^
IT	27.78 ± 2.45 ^c^	2.19 ± 0.23 ^c^	1.2 ± 0.9 ^bc^
IT- PRP	33.65 ± 3.45 ^d^	1.58 ± 0.21 ^b^	1.4 ± 0.82 ^bcd^
IT- PIH	24.81 ± 2.25 ^a^	2.44 ± 0.5 ^ad^	1.7 ± 0.76 ^de^
IT- PRP&PIH	24.51 ± 0.32 ^a^	2.63 ± 0.23 ^ad^	2.08 ± 0.2 ^ae^

Data are presented as mean ± SEM (*n* = 7)

a, b, c, d, and e: According to post-hoc Tukey test which was used to make intergroup comparisons, groups with same superscripts were not significantly different

MDA: Malondialdehyde, SOD: Superoxide dismutase, NOx: Nitrite/nitrate

CON: Healthy control rats

D: Control diabetic rats

IT: Islet transplanted diabetic rats

IT-PRP: Islet transplanted diabetic rats with PRP

IT-PIH: Islet transplanted diabetic rats with PIH

IT-PRP&PIH: Islet transplanted diabetic rats with PRP&PIH

### Stereological parameters

**Fig. 2:**
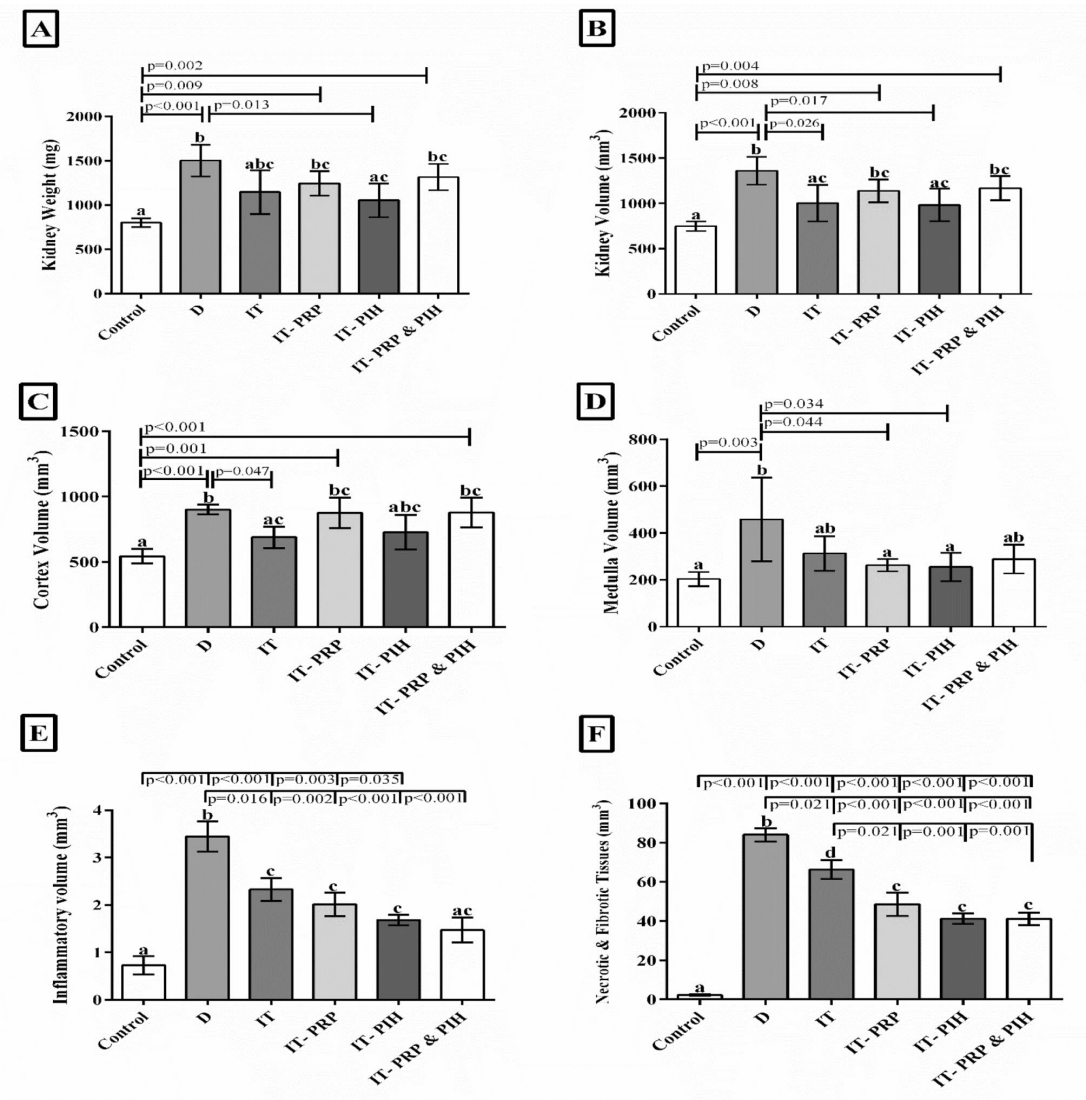
Stereological parameters assessment including (**A**) kidney weight, (**B**) Kidney volume, (**C**) Cortex volume, (**D**) Medulla volume (**E**) Inflammatory volume, and (**F**) Necrotic & Fibrotic tissue volume of biomaterial treated islet-transplanting rats. a, b, and c: According to post-hoc Tukey test which was used to make intergroup comparisons, groups with same superscripts were not significantly different at α = 0.05 (*p* ≥ 0.05). However, dissimilar letters indicate a significant difference (*p* < 0.05). CON: Healthy control rats. D: Control diabetic rats. IT: Islet transplanted diabetic rats. IT-PRP: Islet transplanted diabetic rats with PRP. IT-PIH: Islet transplanted diabetic rats with PIH. IT-PRP&PIH: Islet transplanted diabetic rats with PRP&PIH

The kidneys in D (*p* < 0.001), IT-PRP (*p* = 0.008), and IT–PP & PIH (*p* = 0.002) animals showed a marked increase in their total weight and volume compared with controls. This implies that the PIH-treated islet transplant should significantly reduce the above parameters as compared to the D group (*p* = 0.013) (Fig. 2).

The kidney cortex volume in all diabetic group except IT group was higher than the control group, however; this difference was just remarkable in D (*p* < 0.001), IT-PRP (*p* = 0.009), and IT-PRP&PIH (*P* = 0.002) groups. In comparison to D group, this parameter was only significantly lower in IT animals (*p* = 0.047). There was no notable difference among islets transplanting diabetic groups (Fig. 2).

Although the volume of medulla was enhanced in all diabetic groups compared to the control group, it was only significant in the D group (*p* = 0.003)). Biomaterial- treated islet transplantation could significantly decrease medulla volume in IT-PRP (*p* = 0.044) and IT-PIH (*p* = 0.034) groups compare to D animals. There was no significant difference among islet transplanting groups (Fig. 2).

As shown in Fig. 2, glomerulus volume in all diabetic groups except IT and IT-PIH was markedly increased than the control group. A significant decrease in glomerulus volume was observed in IT compared to the D group ((*p* < 0.001). A significant enhancement in biomaterial- treated islet transplanting: IT-PRP (*p* < 0.001) and IT-PIH (*p* = 0.001) groups was observed than IT group.

**Fig. 3:**
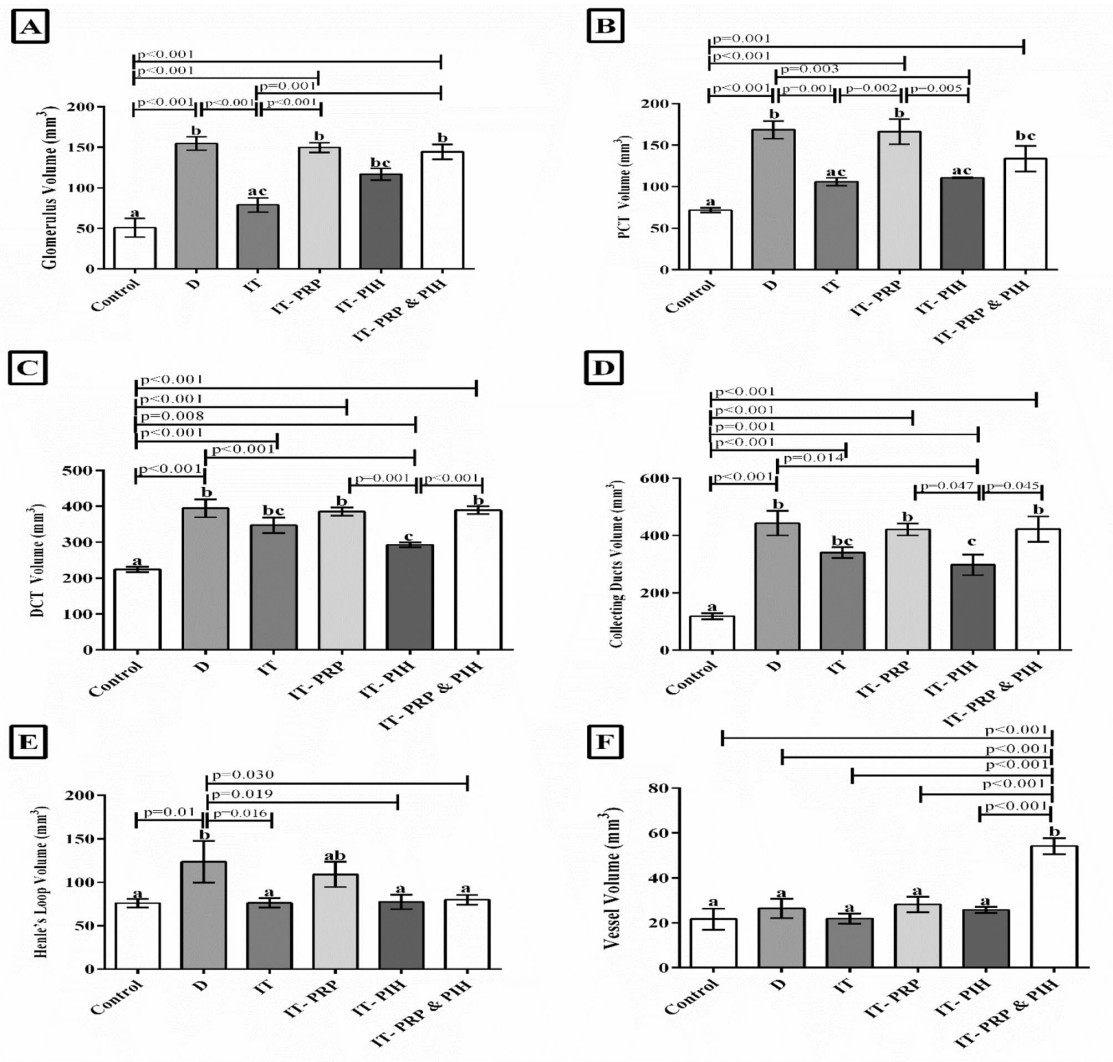
Stereological parameters assessment including (**A**) Glomerulus, (**B**) PCT volume, (**C**) DCT volume, (**D**) Collecting ducts volume (**E**) Henles’ loop volume, and (**F**) Vessel volume of biomaterial treated islet-transplanting rats. PCT: Proximal convoluted tubule; DCT: Distal convoluted tubule; a, b, and c: According to post-hoc Tukey test which was used to make intergroup comparisons, groups with same superscripts were not significantly different at α = 0.05 (*p* ≥ 0.05). However, dissimilar letters indicate a significant difference (*p* < 0.05). CON: Healthy control rats. D: Control diabetic rats. IT: Islet transplanted diabetic rats. IT-PRP: Islet transplanted diabetic rats with PRP. IT-PIH: Islet transplanted diabetic rats with PIH. IT-PRP&PIH: Islet transplanted diabetic rats with PRP&PIH

PCT volume in D, IT-PRP, and IT-PRP&PIH groups was significantly higher compared to control group (*p* < 0.001). This parameter was markedly lower in IT (pp = 0.001), and IT-PIH (*p* = 0.003) than D animals. PCT volume considerably increased in IT-PRP than IT (*p* = 0.002) animals and decreased in IT-PIH than IT-PRP animals (*p* = 0.005) (Fig. 3).

DCT and collecting ducts volume were significantly higher in all diabetic animal compared to control group (*p* ≤ 0.001), and incredibly lower in IT- PIH group than D animals (*p* ≤ 0.001). Among the biomaterial- treated islet transplanting animals, a remarkable increase in these parameters were observed in IT-PRP and IT-PRP&PIH (*p* ≤ 0.001) than IT-PIH group (Fig. 3).

As shown in Fig. 3, Henle’s loop volume was notably higher in D compared to the control group (*p* = 0.01), and significantly lower in IT (*p* = 0.016), IT-PIH (*p* = 0.019), and IT-PRP&PIH (*p* = 0.3) than in the D group.

Except IT-PRP&PIH group, all other diabetic groups showed significantly more inflammatory volume compared to the control group: D and IT (*p* < 0.001), IT-PRP (*p* = 0.003), and IT-PIH (*p* = 0.035). This parameter significantly decreased in IT (*p* = 0.016), IT-PRP (*p* = 0.002), IT-PIH, and IT-PRP&PIH (*p* < 0.001), groups than D animals (Fig. 2).

The necrosis and fibrosis tissue volume of the kidney in the diabetic groups has increased significantly compared to the control group (*p* < 0.001). Among diabetic groups, this parameter was significantly lower in all islets transplanting animals: IT (*p* = 0.21), IT-PRP, IT-PIH, and IT-PRP&PIH (*p* < 0.001) than in D animals. In Comparison between islets transplanting animals, necrotic and fibrotic tissue was markedly lower in the biomaterial- treated islets IT-PRP (*p* = 0.21), IT-PIH, and IT-PRP&PIH (*p* = 0.001) groups than IT group (Fig. 3).

The vessel volume was markedly higher in IT-PRP&PIH compare to the control group, D group, IT animals, and two other biomaterial treated islets IT-PRP, and IT-PIH groups (*p* < 0.001) (Fig. 3).

**Fig. 4:**
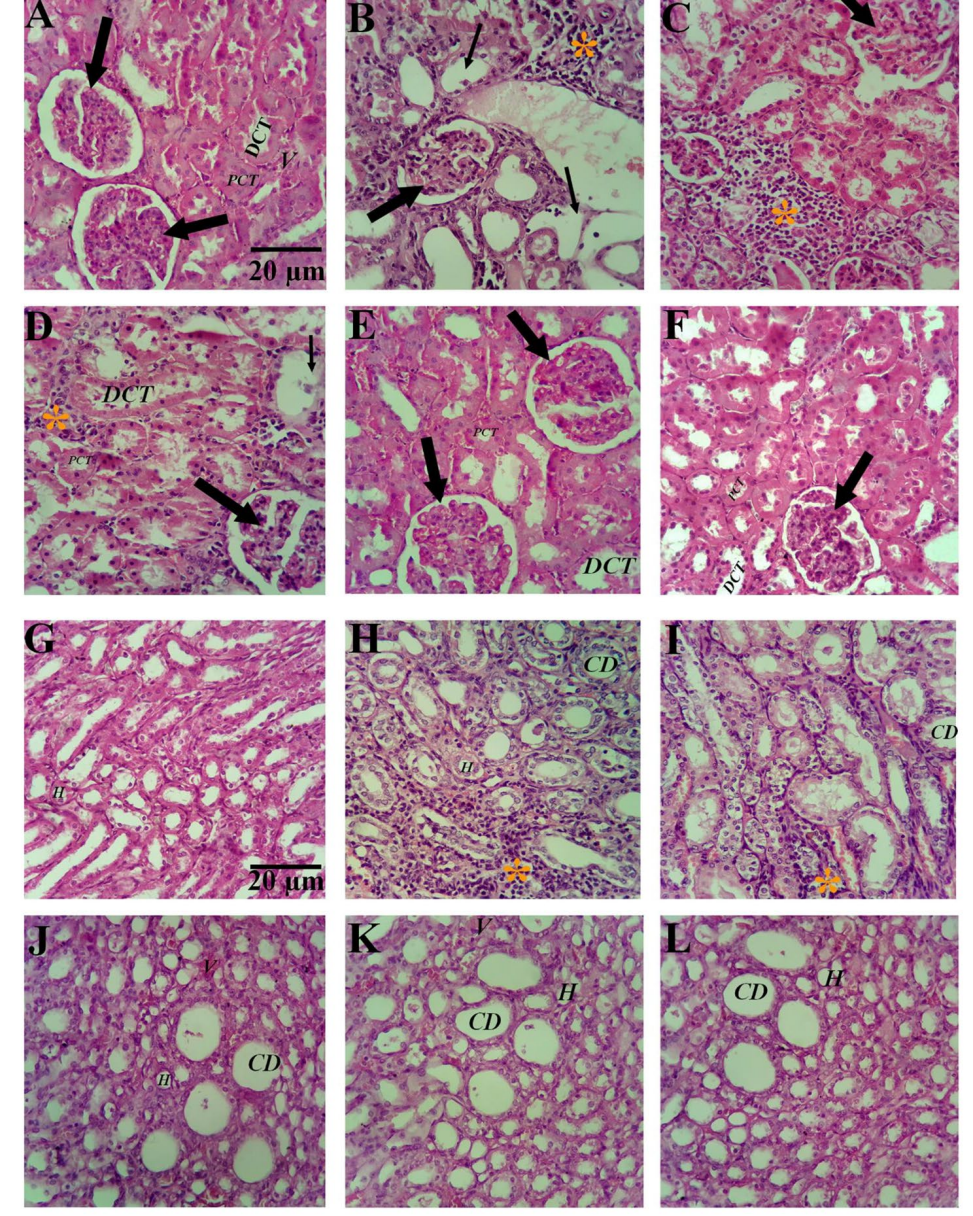
Renal histological microscopic image in different groups (“Masson’s trichrome” and “Hematoxylin eosin” dyes stain×400). No histopathological changes were shown in the control group (A & G). In the Diabetic group (B& H) an increment in inflammation (orang Star sign), interstitial hemorrhage, and Hyaline casts, necrotic tissues (thin arrow) was observed multifocally from the cortex to the inner medulla along the nephrons. On the other hand, the destruction and deformation of the proximal and distal convoluted tubules were observed in some places in the all diabetic group. The Image of IT group (C & I) indicated an enhancement of inflammation, and Hyaline casts, and necrotic tissues were observed multifocally from the cortex to the inner medulla along nephrons. The IT-PRP group (D & J) showed no significant changes compared to the IT group (Except for glomerular, pct volumes and necrotic tissues), while the IT-PIH and group (E & K) showed significant amelioration in the diabetic-induced structural damages such as inflammation, and deformation of proximal and distal convoluted tubules in renal tissue. There was no significant difference in inflammation area in the IT-PRP&PIH group (F & L) compared to the control group. PCT: proximal convoluted tubule; DCT: Distal convoluted tubule; CD: Collecting ductus; H:Henle’s loop; V: Vessel. The thick arrow indicates the glomeruli of the kidney tissue. CON: Healthy control rats. D: Control diabetic rats. IT: Islet transplanted diabetic rats. IT-PRP: Islet transplanted diabetic rats with PRP. IT-PIH: Islet transplanted diabetic rats with PIH. IT-PRP&PIH: Islet transplanted diabetic rats with PRP&PIH

Histological microscopic images and kidney histopathological changes in the different experimental groups which mentioned above are shown in Fig. 4 (A-L).

## Discussion

Hyperglycemia is involved in the development of diabetic nephropathy both directly and indirectly through oxidative stress [4]. Results of previous studies showed that transplantation of islets incubated with biomaterials (PRP&PIH) could significantly decrease blood glucose level [18, 19]. According to this fact that controlling blood glucose has a pivotal role in treating DN. In the present study, we investigated the efficacy of islets co-transplantation with safe, cost-effective, and minimal immune response biomaterials on the treatment or reverse of diabetic nephropathy. Similar studies to this has not been done.

Our results indicated that these biomaterials, either alone or in combination, induced different effects on the functional and structural changes of the kidney in diabetic rats. These effects may be partly due to the presence of different growth factors in the biomaterials used. Results clearly demonstrated the advantages and disadvantages of transplanting islets that were previously incubated with PRP and/or PIH on diabetic nephropathy.

After a remarkable reduction in hyperglycemia following islets transplantation, kidney function improved compared to the diabetic control group, which increased serum albumin, and decreased BUN concentration. The stereological changes evaluation showed no significant differences in several parameters including kidney weight, total kidney volume, and volume of the cortex, medulla, glomerulus, inflammatory, and necrotic & fibrotic tissue among biomaterials-treated islets transplanting groups. While a significant increase in PCT volume in IT-PRP, DCT, and CD volume in IT-PRP and IT-PRP&PIH compared to IT-PIH group, as well as significant increase of vessel volume in IT-PRP&PIH group compared to IT-PRP and IT-PIH was observed. These functional and structural changes in different biomaterials treated islets transplanting diabetic groups was partly related to the decreased blood glucose level and partly due to the used biomaterials.

Although islet transplantation reduced hyperglycemia, it was still higher than in the healthy control group. A study showed that the higher glucose level induced the structural changes in the kidney, such as an increase in the volume of the cortex, medulla, a glomerulus, and a decrease in the number of glomeruli, some of these changes were also observed in the present study. PRP has a wide range of growth factors, including IGF-1, VEGF, HGF, EGF, PDGF, and TGF- β. It has been observed that IGF increases the growth of human mesangial cells [23], stimulates the production of proteoglycans by human embryonic mesangial cells [24], and vascular endothelial cells [25], in in vitro conditions, as well as excessive production of this growth factor in NOD mice mesangial cells causes the accumulation of ECM [26], which demonstrates that IGF is the main factor in the progress of glomerulopathy. Although in the present study, the growth of mesangial cells and the accumulation of the ECM were not measured (that is our study limitation), the less significant increase in serum Alb concentration in IT-PRP animals than in two other biomaterial-treated islets groups are partly may be due to IGF which induces glomerulopathy and partly is due to the presence of HGF/ TGF- β. Hepatocyte growth factor plays a role in kidney regeneration and preservation. During the development of chronic kidney disease or fibrosis, the expression of HGF reaches lower level than normal. In addition, a decrease in the expression of HGF compared to TGF- β is related to the progression of kidneys disease [27]. The existence of HGF in PRP and PIH justifies the better regeneration process of kidney in these groups. In PIH, there is only HGF, not TGF- β and in PRP&PIH, the concentration of HGF is higher than TGF- β, which causes a significant increase in kidney function, so the albumin level in IT-PRP&PIH and IT-PIH groups was higher compared to IT-PRP. In the IT-PRP group, due to the kidney disease, HGF and TGF- β expression by the kidney decreased and increased, respectively. Despite the presence of HGF in PRP, the existence of TGF- β in PRP causes less significant improvement in kidney function than in the IT-PRP&PIH and IT-PIH groups. The improvement of kidney function in IT animals compared to diabetic control animals was because of reducing blood glucose level.

The kidney is one site of TGF- β generation and activation. Also, the effects of this GF on different cell type of glomerulus and proximal tubules have been observed [28, 29]. It has been shown that TGF- β is partially involved in the progression of diabetic nephropathy in vitro condition. Another study described that activation of TGF-β causes an induction of ECM formation and fibrosis [30]. On the other hand, hyperglycemia enhances the expression level of TGF-β mRNA in mesangial and proximal cells in vitro conditions [31–33]. These findings justify the higher necrotic and fibrotic tissue volume of IT-PRP animals compared to IT-PIH and IT-PRP&PIH groups. Also, Kanwae et al., reported that high glucose concentrations induced TGF- β mRNA enhancement in combination with reducing activity matrix-degrading metalloproteinase [34], resulting in ECM aggregation [35].

The presence of VEGF and its receptors in different parts of the kidney, such as glomerular epithelial cells [36], podocytes [36, 37], distal tubule, and collecting duct [37, 38] has been confirmed. It has been demonstrated that hyperglycemia stimulates the expression level of VEGF in vascular smooth muscle cells [39]. Also, a wide range of GFs and cytokines like TGF-β [40] and PDGF [41] have been observed to prompt the generation of VEGF in various cell types outside the kidney [42] that may be releases in blood circulation and bound to their receptors in the kidney, resulting in deteriorated nephropathy. VEGF is one of the main factors that stimulates angiogenesis, and proliferation of endothelial cells [19]. According to this, the result of the present study showed that IT-PRP&PIH group, which islet incubated in media including a high concentration of different GFs either directly or indirectly by stimulating the production of VEGF, causes more angiogenesis. There are more blood vessels in IT-PRP&PIH group, which is significantly higher and different from other diabetic groups as well as the healthy control group. Also, as mentioned above, higher vessel volume in the IT-PRP and D groups are because of the presence of VEGF in PRP- supplemented media, and blood hyperglycemia, respectively.

On the other hand, VEGF activity is dependent on the production of endothelial nitric oxide (eNO) [43]. Coupling VEGF with eNO can improve or worsen kidney function depending on whether it has a pro-inflammatory or anti-inflammatory effect [44]. In this study, VEGF showed anti- inflammatory effect, so in IT-PRP&PIH group, due to the high level of VEGF and its coupling with eNO, led to a reduction in the volume of inflammation compared to other biomaterial treated islets transplanting groups. In the confirmation, the results showed that decrease eNO and VEGF have a direct relation with the increased inflammatory volume.

## Conclusion

Our findings suggest that treated islets transplanting could improve diabetic nephropathy. Improvement of oxidative stress followed by controlling glucose levels and the effects of some growth factors present in these biomaterials can be considered a capable underlying mechanism of ameliorating inflammatory, necrotic, and fibrotic tissue volume.

## Data Availability

Data are available thought sending email to correspond authors.
